# Challenging Empathic Deficit Models of Autism Through Responses to Serious Literature

**DOI:** 10.3389/fpsyg.2022.828603

**Published:** 2022-02-10

**Authors:** Melissa Chapple, Philip Davis, Josie Billington, Sophie Williams, Rhiannon Corcoran

**Affiliations:** ^1^Deparment of Primary Care and Mental Health, School of Psychology, University of Liverpool, Liverpool, United Kingdom; ^2^Centre for Research into Reading, Literature and Society, University of Liverpool, Liverpool, United Kingdom

**Keywords:** autism, empathy, literary fiction, creative writing, neurodiversity

## Abstract

Dominant theoretical models of autism and resultant research enquiries have long centered upon an assumed autism-specific empathy deficit. Associated empirical research has largely relied upon cognitive tests that lack ecological validity and associate empathic skill with heuristic-based judgments from limited snapshots of social information. This artificial separation of thought and feeling fails to replicate the complexity of real-world empathy, and places socially tentative individuals at a relative disadvantage. The present study aimed to qualitatively explore how serious literary fiction, through its ability to simulate real-world empathic response, could therefore enable more ecologically valid insights into the comparative empathic experiences of autistic and non-autistic individuals. Eight autistic and seven non-autistic participants read *Of Mice and Men* for six days while completing a semi-structured reflective diary. On finishing the book, participants were asked to engage in three creative writing tasks that encouraged reflective thinking across the novel. Thematic and literary analysis of the diary reflections and writing tasks revealed three main themes (1) Distance from the Novel; (2) Mobility of Response; (3) Re-Creating Literature. Findings demonstrated the usefulness of serious literature as a research tool for comparing the empathic experiences of autistic and non-autistic individuals. Specifically, autistic individuals often showed enhanced socio-empathic understandings of the literature with no empathy deficits when compared to non-autistic participants.

## Introduction

There is currently no agreed consensus for defining ‘autism' as a concept. However, the term generally refers to a form of human neurocognition that is developmental in nature and which results in divergent socio-cognitive processing styles (Fletcher-Watson and Happé, 2019; Milton, 2020). While there is an increasing move toward understanding autistic people through explorations of their nuanced human experiences (Wright et al., 2014), the medical model of disability continues to largely dominate how society thinks about autism and autistic people (Waltz, 2013; Kapp, 2020; Chapple and Worsley, 2021). Although medical categorisations of autism are consistently evolving, the model typically focusses on socio-communicative difficulties, repetitive behavioral patterns and restricted interests (Murray et al., 2005; American Psychiatric Association, 2013; Kapp, 2020). While medical diagnoses offer a route for self-discovery and access to formal support (Mogensen and Mason, 2015; Leedham et al., 2020), the treatment of human neurocognitive diversity in much the same way as physiological disease risks overlooking individualised human experiences (Kinderman et al., 2013). As a result of dominant medical framings, autism research has long over-focused on what autistic people lack (Murray, 2020). In this way, autistic people are positioned as being in need of ‘fixing' in order to align their behaviors with those typically expected within mainstream cultures (Milton, 2012; Waltz, 2013). As a consequence of these views, the autistic community have been denied agency in shaping their own narratives and influencing how they are viewed within society (Milton, 2012; Yergeau, 2013; Fletcher-Watson and Bird, 2020). Instead, dominant theoretical models and subsequent empirical enquiries often employ and further develop societal understandings of autism that reduce and stereotype the nature of autistic experiences (Chapple and Worsley, 2021).

In particular, dominant theories of autism including the weak central coherence (WCC; Happé, 1999), mindblindness (Baron-Cohen, 1997) and empathising-systemising (E-S; Baron-Cohen, 2002, 2009) theories have broadly sought to identify key autism deficits. Specifically, the WCC theory assumes a global processing deficit amongst autistic individuals, believed to result in increased attention to fine detail alongside resultant difficulties around integrating information within a wider context (Happé, 1999; Hill, 2004). In relation to social processing, autistic cognition is then positioned as problematic against an assumed need within everyday social situations to quickly integrate facets of social information into a coherent whole (Happé, 1999; Baron-Cohen, 2009). By contrast, the mindbliness theory (Baron-Cohen, 1997) proposes that autistic individuals experience profound difficulties in representing and attributing mental states to others, otherwise known as theory of mind (ToM; Premack and Woodruff, 1978; Reniers et al., 2011). While these two theories focus on different aspects of autistic cognition, the E-S theory largely combines the underlying ideas of the two approaches (Baron-Cohen, 2009). Specifically, the original E-S theory positioned autistic individuals as broadly less empathic than their non-autistic peers (Baron-Cohen, 2009). Instead, autistic people are argued to process information in a more systematic way, exploring regularities to extract predictable rules (Baron-Cohen, 2009). This systematic approach to learning is argued to be too rote-based to be applicable to the spontaneity of everyday socio-emotional contexts, resulting in broad empathic difficulties (Baron-Cohen, 2009). As a result, autistic individuals have been argued to implement extreme egocentrism, attributing their own mental states to others regardless of contextual information or similarities to self (Lombardo and Baron-Cohen, 2011; Bodner et al., 2015; Ripley, 2015). It is these assumptions of reduced empathic capacity in particular that risk undermining the core human experiences of autistic people (Yergeau, 2013; Fletcher-Watson and Bird, 2020).

Furthermore, these deficit-based assumptions have left a lasting impact, with a resultant, long-standing focus on researching autism-specific empathy deficits (Peterson et al., 2005; White et al., 2009; Song et al., 2019). While empathy as a term is often used inter-changeably across differing concepts, it can broadly be defined as the ability to recognise, share and respond to the feelings of others (Shamay-Tsoory et al., 2009; Fletcher-Watson and Bird, 2020). However, definitions such as these are argued to be specific to affective empathy (Shamay-Tsoory et al., 2009; Smith, 2009), with ToM or ‘cognitive empathy' believed to exist as a separate construct (Shamay-Tsoory et al., 2009; Reniers et al., 2011). Here, affective empathy then refers to the related ability to vicariously experience the emotional states of others (Reniers et al., 2011). With particular influence from the mindblindness theory (Baron-Cohen, 1997), research into assumed empathy deficits amongst autistic individuals has largely focussed on cognitive empathy (Shamay-Tsoory et al., 2009; Smith, 2009). Research into cognitive empathy deficits has concluded that autistic people are impaired in the recognition of complex but not simple emotional states (Icht et al., 2021); are less accurate at inferring emotion from both static and dynamic faces (Rigby et al., 2018); and perform significantly worse than non-autistic individuals on multiple ToM tests (Dziobek et al., 2006). However, these studies often implement standardised ToM tests which rely on fast-paced assumptions to infer in-depth human feelings from limited snapshots of information (Baron-Cohen et al., 2001a; Dziobek et al., 2006). As a result, careful and complex evaluations of mental states would result in unfavorable scoring on such tests. It is these complex considerations that are more reflective of real-world empathy, where affective and cognitive empathic responses cannot be separated so easily into unrelated concepts and instead co-occur in real time (Fletcher-Watson and Bird, 2020).

Additionally, these deficit-based approaches overlook the bi-directional nature of social communication within any given social pair (Milton et al., 2018). Instead, deficit models place an assumption of blame onto autistic individuals when social difficulties arise (Milton, 2012; Chown, 2014). One theory that seeks to address the two-way nature of socio-communicative difficulties is Milton's (2012) double empathy problem. The double empathy problem reframes ToM deficits as an issue of reciprocity and mutuality between individuals within a given socio-communicative exchange (Milton, 2012; Milton et al., 2018). While a lack of mutuality can arise for any two individuals, Milton (2012) suggests that the differing social realities of autistic and non-autistic individuals make breakdowns in communication more likely. Therefore, it is proposed that non-autistic individuals are at least equally likely to misjudge the mental states and feelings of autistic individuals (Milton, 2012; Chown, 2014), an assumption well-supported by empirical research (Brewer et al., 2016; Edey et al., 2016; Sheppard et al., 2016; Heasman and Gillespie, 2019; Crompton et al., 2020b). Furthermore, Milton (2012) opposes the view that autistic individuals fail to incorporate context, highlighting that context is created within a particular exchange. This assumption is supported by findings that when interacting together, autistic individuals experience increased mutuality, resulting in increased social comfort (Crompton et al., 2020a; Morrison et al., 2020); better communicative understandings (Heasman and Gillespie, 2018; Crompton et al., 2020a); and an increased willingness to overcome initial negative impressions (DeBrabander et al., 2019). However, with non-autistic individuals being the majority group, their increased likelihood for experiencing mutuality during social exchanges results in assumptions of pre-determined norms amongst peers (Milton, 2012). It is these assumptions of pre-set social etiquette and understandings that position different Others, such as autistic individuals, as being defective in some way (Milton, 2012; Chown, 2014).

Furthermore, while the double empathy problem is well-supported by research, the related assumption that autistic individuals may have a better understanding of society than non-autistic individuals (Milton, 2012; Chown, 2014) has largely been overlooked. Specifically, it is suggested that autistic individuals are more likely to take time in developing common ground and understanding different Others as a result of being more experienced in navigating a lack of mutuality (Milton, 2012, 2020; Chown, 2014). In this way, autistic individuals may be more likely to work to sensitively and empathically overcome socio-communicative breakdowns rather than drawing quick conclusions based upon assumed pre-existing mutuality (Milton, 2012; Chown, 2014; DeBrabander et al., 2019; Chapple et al., 2021b). Autistic writer Joanne Limburg (2021) expands upon this assumption by arguing that dehumanised individuals, such as those who are autistic, are forced to think about the ways in which modern society is constructed, giving them deeper understandings of the social world. Therefore, autistic individuals may avoid assumptions of pre-existing social norms to consider the feelings and perspectives of different Others in ways that remain open to the complexity of individual experiences (Lesser and Murray, 2020). This is supported by research findings that autistic individuals are more socially tentative, requiring more time and care at the expense of fast-paced judgements that rely on immediate contextual cues alone (Capps et al., 1992). Therefore, what has previously been framed as difficulties with contextual consideration becomes re-framed as a potential advantage in remaining open to emergent social information (Lesser and Murray, 2020). As a result, autistic people may go beyond what is known immediately to tailor their social and affective responses to each individual social encounter empathically (Lesser and Murray, 2020). These assumptions are further expanded upon by the theory of monotropism (Murray et al., 2005), which seeks to expand upon the WCC through a less pathologised approach (Murray, 2020). Specifically, monotropism suggests that autistic individuals have narrow interest systems that direct and sustain attention toward nuanced topics of interest (Murray et al., 2005). While largely similar to the WCC, monotropism does not assume a broader resultant deficit in the integration of information at the detriment of social experience. Instead, the theory draws attention to the depth of feeling experienced by autistic individuals as a result of highly-focussed interest systems (Murray, 2020). However, the theory still positions these advantages as existing at the expense of understanding social breadth, or the ‘modeling of other minds' (Lesser and Murray, 2020; Murray, 2020).

While these open and complex empathic understandings are difficult to research with standardised experimental tests (Fletcher-Watson and Bird, 2020), the exploration of reflection in response to fictional texts offers a unique way to explore empathic understandings within an ecologically valid context (Chapple et al., 2021b). Specifically, fiction is argued to simulate the real social world (Mar and Oatley, 2008; Waytz et al., 2015; Oatley, 2016), where readers can embody character perspectives and feelings to achieve felt empathy (Mumper and Gerrig, 2019). While the use of personal thought and feeling to understand, appreciate and experience a text could be criticised as egocentric (Lombardo and Baron-Cohen, 2011), fiction encourages an overcoming of social pressures and conformity in a way that moves readers away from default or rigid ways of thinking (O'Sullivan et al., 2015; Davis, 2020; Davis and Magee, 2020). Furthermore, fiction is argued to take readers beyond the process of imposing their own thoughts and feelings onto others, instead encouraging a mutual feeling together with the text and the minds within it (Mumper and Gerrig, 2019). Not only does fiction evoke feeling within a text in this way, but also requires co-occurring perspective-taking with the minds that are being represented (Zunshine, 2011). Specifically, readers are required to access the minds of characters through the mind of the author, with those minds ultimately being processed through a reader's own personal perspective (Zunshine, 2011). As a result, the distinction between affective and cognitive empathy becomes artificial while reading, with both thought and feeling working fluidly together in a way that reflects real-world empathy (Koopman, 2016; Fletcher-Watson and Bird, 2020). Therefore, it is argued that fiction acts like a flight simulator, providing the opportunity to engage with multiple minds across social experiences (Mar and Oatley, 2008; Mumper and Gerrig, 2019). This has been supported by research findings which indicate that engagement with fiction may enhance ToM performance and wider empathic capacity (Mar et al., 2009; Bal and Veltkamp, 2013; Kidd and Castano, 2013). Additionally, empathic feeling can be enhanced while reading, due to the ability to feel with different Others without negative social or personal consequence (Koopman and Hakemulder, 2015; Koopman, 2016). Therefore, fiction is thought to be of social benefit to its readers, enhancing a reader's empathic capacity for different Others by providing opportunities for embodied reflection through a pluralism of simulated social experience (Oatley, 2002, 2016; Bal and Veltkamp, 2013).

Furthermore, it is thought that serious literary fiction is particularly advantageous in promoting this empathic embodiment of different Others within a text (Mar and Oatley, 2008; Koopman and Hakemulder, 2015; Davis, 2020; Davis and Magee, 2020). Here, serious literature specifically refers to texts that engage with significant human situations, subsequently enabling its readers to do the same (Koopman and Hakemulder, 2015; Davis and Magee, 2020). While it has been argued that different Others are essentially unknown and unknowable (Levinas, 1969), the mirroring of real human situations within literature is argued to result in imaginative feelings with the characters, situations and feelings within it (Davis, 2020). Therefore, it is argued that serious literature enables readers to form more in-depth understandings of human existence through imaginative feeling with other minds (Koopman, 2016; Davis, 2020). This imaginative capacity to treat literary characters as real and employ their point of view is believed to be true across narrative settings, regardless of realism (Anderson et al., 2019). Specifically, it is argued that it is the words which hold the potential of powerful and active beings in themselves (Erdman, 1978). In this way, the powerful language within serious literature encourages readers away from processing in easy, heuristically-driven, automatic ways that avoid ambiguity in order to reach quick conclusions (Djikic et al., 2013; O'Sullivan et al., 2015; Davis, 2020). Instead, literature encourages readers to hold onto what feels like emotionally salient moments of a text, also known as close reading, as opposed to information-scanning (Davis, 2013; Wolf, 2018). In this way, the close reading encouraged by serious literature allows for slower reflections to explore the embedded complexities of social realities (Mar and Oatley, 2008; Koopman and Hakemulder, 2015; O'Sullivan et al., 2015; Koopman, 2016). Furthermore, this holding of ambiguity and feeling within literature reflects a suspended judgment in which empathic feelings are enhanced, because the ambiguity of a text means readers cannot rely on schematic inferences (Koopman and Hakemulder, 2015). Instead, readers are moved toward new ways of thinking that are receptive and flexible, enabling sudden re-considerations in real time, in direct response to emergent information (Davis, 2020; Davis and Magee, 2020). These movements evoked by a text are argued to be more powerful when experienced through adversity (Strick and Van Soolingen, 2018; Davis, 2020). It is therefore assumed that texts dealing with adversity may be more moving, prompting new, more careful ways of thinking about different minds (Strick and Van Soolingen, 2018; Davis, 2020).

While some readers may remain on the surface of serious literature, struggling to get within it, those who experience what Limburg (2021) calls undifferentiation show the true advantages of literary reading (Barnes, 2018; Davis, 2020; Davis and Magee, 2020). During this process, it is argued that moving parts of a passage become part of the reader, while simultaneously remaining part of the text and the author who wrote it, all at the same time (Barnes, 2018). In this way, it becomes necessary for readers to re-write serious literature in the act of reading (Barthes, 1969, as cited by Muldoon, 2021). This is to say that readers of serious literature are not simply reading, rather they are mentally ‘doing' the literature in the process of reading (Barthes, 1969; as cited by Muldoon, 2021). Therefore, the careful, slower processing of thought and feeling that is commonly observed amongst autistic individuals (Capps et al., 1992; Lesser and Murray, 2020) could make them more ‘literary' readers. In particular, those who deal with adversity in their daily lives, such as autistic individuals, may be more powerfully moved by serious literature (Strick and Van Soolingen, 2018; Davis, 2020) and prompted to further reconstruct their views on societal construction (Limburg, 2021). This means that the utilisation of serious literature within autism research offers a way to more accurately compare the empathic experiences of autistic and non-autistic individuals. Furthermore, as serious literature prevents fast-paced assumptions based on schematic inference (Mar and Oatley, 2008; Koopman and Hakemulder, 2015; O'Sullivan et al., 2015; Koopman, 2016) it might then prompt non-autistic readers to think more empathically about minds different from their own. Therefore, reading may serve to overcome the positioning of different minds as defective (Chapple et al., 2021b).

However, as research enquiry into the value of fiction for autistic readers has largely been restricted by deficit-based assumptions, it has been assumed that autistic individuals lack the socio-cognitive capacity to contemplate and enjoy fiction (Baron-Cohen, 1997, 2009). Instead, it has been assumed that autistic individuals would prefer the systematic nature of factual non-fiction (Baron-Cohen, 2009; Barnes, 2012). However, recent findings have contradicted dominant assumptions, showing instead that autistic individuals across age groups do engage with fiction and literary non-fiction (Barnes, 2012; Davidson and Ellis Weismer, 2018; Armstrong et al., 2019; Chapple et al., 2021a). Additionally, findings show that when asked about their experiences of reading, autistic participants report examples of felt empathy for fictional characters and book authors themselves (Chapple et al., 2021a). However, little is known about the way in which autistic individuals would engage with serious literature, and how this might compare to non-autistic individuals. Further research is also needed to examine assumptions of in-depth processing amongst autistic individuals at the expense of modeling other minds (Happé, 1999; Murray et al., 2005). While this in-depth local processing may enhance autistic readers' ability to hold in mind moving passages, monotropism assumptions indicate that their wider considerations of social construction may be limited.

To address this evidence gap, the current study qualitatively explores how autistic adults engage with serious literature in comparison to non-autistic adults. Specifically, participants read *Of Mice and Men* (Steinbeck, 1937) while completing a semi-structured diary that prompted daily reflections on the novel and its characters, with some creative writing tasks upon completion of the novel. The novel was chosen primarily due to its complex exploration of stigma and Othering toward and within groups of disabled characters with inter-sectional marginalized identities (Chapple et al., 2021b). Additionally, the novel was chosen due to the relative ease of initial access to the minds within realistic texts for inexperienced readers. This was advantageous for the current project, where the literary exposure of the participants was unknown, and due to a current lack of research into textual factors that enhance empathic feeling amongst autistic participants and within a double empathy paradigm. Furthermore, the representation of disability within the novel encourages readers to embody feelings of adversity, allowing for the exploration of movement in autistic compared to non-autistic readers (Strick and Van Soolingen, 2018; Davis, 2020). The current study was part of a wider research project, where participants later went on to discuss the novel to explore resultant double empathy understandings between autistic and non-autistic readers (Chapple et al., 2021b). For the present study, the aim was to address two research questions: ‘can reflections on a piece of serious literature offer direct evidence that autistic adults engage empathically with complex characters and social content?' and ‘is there evidence that autistic adults read in a more ‘literary' way than non-autistic readers?' Based on suggestions that autistic individuals are more socially tentative (Capps et al., 1992; Murray et al., 2005; Milton, 2012, 2020; Chown, 2014; Lesser and Murray, 2020), it was predicted that the autistic participants would engage empathically with the novel and read in a more literary way.

## Methods

### Participants

Participants were recruited through social media and University advertisements. A total of 27 participants took part in the initial screening process for inclusion in the study. Eight autistic and 8 non-autistic participants were invited to take part in the research. However, 1 non-autistic participant dropped out of the study and was not replaced due to having achieved data saturation within the material collected from the remaining 7 non-autistic participants. Of the remaining 11 participants who were screened, 2 (1 autistic) dropped out of the study early on in the recruitment process. Contact details of the remaining 9 participants were kept on file for another research project. Inclusion criteria included being 18 or over, having proficient English language skills, and scoring an estimated Wechsler Adult Intelligence Scale (WAIS) IQ score of 90 or above as assessed by the Quick Test (QT; Ammons and Ammons, 1962). For autistic adults who did not have an official diagnosis (e.g., referred for assessment or self-identified), there was an exclusion criterion of scoring below 32 (the suggested cut off for autism) on the AQ (Baron-Cohen et al., 2001b). Undiagnosed autistic participants were included to take account of accurate gender representation due to the longstanding underdiagnosis of women (Cooper et al., 2018; Fletcher-Watson and Happé, 2019). Non-autistic participants had an additional exclusion criterion of scoring over 32 on the AQ.

Overall, fifteen participants provided data for this research study (see Tables 1, 2 for demographics). Eight were autistic (male *N* = 4; female *N* = 4) aged 19–48 (*M* = 30.75, SD = 9.22) and seven were non-autistic (male *N* = 3; female *N* = 4) aged 23–56 (*M* = 38.57, SD = 13.10). The study was approved by the University of Liverpool Research Ethics Committee.

**Table 1:** Participant AQ and IQ scores [mean (±SD)].

	**AQ^***a***^**	**Estimated IQ^***b***^**
		**(WAIS equivalent)**
Autistic	40.50 (6.57)	100.00 (5.13)
Non-autistic	11.71 (4.92)	101.14 (6.09)

*AQ, Autism quotient; QT, Quick test; WAIS, Wechsler Abbreviated Scale of Intelligence*.

a *AQ scores*

b *IQ assessed by the QT*.

**Table 2:** Participant demographics.

**Participant no**.	**Age**	**Gender**	**AQ^***a***^**	**IQ^***b***^ (WAIS equivalent)**	**Level of education completed**	**Neurotype**
1	29	Male	42	96	GCSE	Autistic: diagnosed
6	46	Male	17	90	A Level	Non-autistic
7	23	Male	10	100	Masters	Non-autistic
8	26	Female	12	102	Bachelors	Non-autistic
9	33	Female	12	100	Doctoral Training	Non-autistic
10	33	Male	13	108	Foundation or Diploma	Non-autistic
11	48	Female	44	108	Doctoral Training	Autistic: diagnosed
14	53	Female	16	100	Masters	Non-autistic
17	56	Female	2	108	Bachelors	Non-autistic
18	25	Male	44	98	Masters	Autistic: diagnosed
20	19	Female	30	92	A Level	Autistic: diagnosed
21	28	Female	48	104	Masters	Autistic: self-diagnosed
23	33	Female	46	104	Bachelors	Autistic: diagnosed
25	39	Male	38	100	Masters	Autistic: diagnosed
27	24	Male	32	98	Bachelors	Autistic: diagnosed

*AQ, Autism quotient; QT, Quick test; WAIS, Wechsler Abbreviated Scale of Intelligence*.

a *AQ scores*

b *IQ assessed by the QT*.

### Screening Measures

A demographics questionnaire asked for participants' age, gender, and highest completed qualification. Eligibility questions were asked at this stage.

#### The Autism Quotient (AQ)

The AQ (Baron-Cohen et al., 2001a) is a 50-item questionnaire that uses statements to elicit a score that reflects autistic traits in clinical and non-clinical samples. The AQ was used to assess the number of self-reported autistic traits in both samples.

#### The Quick Test (QT)

A single 50-item version of the QT (Ammons and Ammons, 1962) was used. The test involves participants looking at 4 pictures and deciding which picture each word goes best with. Given the age of the QT, the raw test score is converted to a WAIS, not WAIS-R, equivalent IQ. Although not ideal and rather dated, this was considered an adequate method for obtaining a rough estimate of reading comprehension ability for this study where its brevity was an asset and where IQ data was not going to be subjected to further analysis.

### Diary and Interview Measures

#### Participant Diaries

A structured diary was designed for participants to record their thoughts while reading Of Mice and Men (Steinbeck, 1937). The diary was completed for 7 days, the first 6 coincided with reading the book at a rate of one chapter per day. For each chapter, participants were asked 5 questions, questions 1 to 3 were designed to prompt general reflections about narrative events and characters: (1) *What thoughts or feelings did chapter X prompt?* (2) *Do you think the characters in chapter X were realistic?* (3) *Did you like or dislike the characters in chapter X?* Questions 4 and 5 were added based on previous findings that autistic readers think more about author intent (Chapple et al., 2021a) (4) *Did you think about the author when reading chapter X?* (5) *What did you think the author was trying to achieve in chapter X?* On day 7, participants completed 3 writing tasks: (1) writing a letter to a character of choice as either (a) themselves, (b) another character, or (c) the author (2) writing a letter to the author as either (a) themselves, or (b) another character and (3) re-writing the ending as they would have preferred it to have ended. These tasks were included to promote reflection on the overall novel and subsequent perspective taking. Tasks 1 and 2 were based on Green's (2020) letter writing methodology for reflective reading, with task 3 included to explore how participants dealt with the novel's emotionally difficult ending.

### Procedure

Potential participants completed a screening process via Qualtrics that included the informed consent procedure, a demographic questionnaire, the QT and the AQ. Participants who screened out or did not leave an email address for contact had their data removed. Informed consent was obtained at two points (1) before screening and (2) before commencement of the diary task. At each stage, participants were provided with both a university standard information sheet as well as an easy-read version that avoided complicated explanations and used clear photographs and text segmentation. Both information sheets encouraged participants to contact the first or fifth author for more information at each stage of the process. The informed consent procedure included the disclosure of participant demographics for data processing.

Upon obtaining informed consent, all participants were provided with either a physical or digital copy of Of Mice and Men (Steinbeck, 1937) and a copy of the diary template. The diary template contained a page of clear instructions with warnings about the sensitive content in the novel. Participants were asked to read one chapter per day for 6 days and to complete the writing tasks on the 7th day and, as far as possible, to stick to the 7-day schedule laid out in the instructions. Upon return of the completed diary, participants were reimbursed £10 for their time in the form of either a £10 Amazon voucher or as cash.

### Analysis

Thematic analysis was chosen to analyse the data deductively, exploring surface-level psychological themes (Clarke and Braun, 2014). A form of literary close reading analysis (Billington et al., 2019) was implemented alongside thematic analysis to inductively explore the data for evidence of deeper psychological shifts within participants as a result of reading. This analysis relies on participant language as ‘the main point of access to moments of subtle mental change' that gives access to the ‘imprints' of reading (Kaszynska, 2015). These qualitative analyses combined to ensure a deep and rich exploration of the data. Analysis stages were as follows:

1) The first author read all participant diaries to achieve data immersion.
2) The first and fourth authors separately coded all of the autistic participant diaries using thematic analysis. All authors then met regularly to deliberate on initial themes until agreement was met. The first author then coded the non-autistic participants, organising codes into the same themes agreed for the autistic participant diaries. The fourth author read over the resultant codes and agreed on the interpretation of the non-autistic diaries.
3) The first author highlighted moments of literary interest in 8 diaries (6 autistic) and sent the diaries to the second and third authors who are trained in close literary reading analysis. The second author read all 8 diaries for immersion and highlighted additional important moments of psychological change. The third author read 4 of the diaries (3 autistic), providing additional commentary on areas of interest.
4) The second author decided on key literary themes within the 8 diaries that were analysed. The first and second author then worked together to re-interpret the data until themes from stage 2 and 3 were successfully integrated. These themes were then sent back to the third and fourth authors who agreed with the re-integration.
5) The first author then re-analysed the remaining 7 diaries (2 autistic) and follow-up data using the integrated approach of thematic analysis with close literary reading that had been agreed on in stages 3 and 4.
6) Resulting themes were deliberated by the rest of the team, with theme names and framings adjusted to capture the main elements of significance within the themes.

The first author is an autistic researcher. The fourth author is an autistic adult who took the role of expert by experience. Therefore, all data was analysed from both autistic and non-autistic perspectives.

## Results

All participants experienced times of being invested within the literature as well as times of struggling to become or remain invested in the literature. The final analysis (see Table 3) comprised three themes: (1) distance from the novel (2) mobility of response and (3) re-creating literature. Participant quotes are split by neurotype group (A: autistic, N: non-autistic). Within the participant quotes, words that depict emergent thinking are highlighted in bold.

**Table 3:** Final analysis themes and subthemes.

Distance from the novel	Difficulties with understanding and immersion
	Emotional distancing
	Socio-political and historical representation
Mobility of response	Active responding
	Thinking aloud and thinking along
	Involuntary feeling for
	More than one thing at a time
	Involvement in a character
Re-creating literature	Emotional depth
	Responsive language changes

### Distance From the Novel

#### Difficulties With Understanding and Immersion

All participants experienced moments while reading the novel when they struggled to ‘get inside' the text, instead evaluating the novel's characters and events from a distance. This distance was largely created as a result of participants' difficulties, across both groups, in understanding the culture and metaphors embedded in the novel, often as a result of what seemed an unfamiliar language:

*(P21A) [in response to “s'pose Curley jumps a big guy an' licks him”] ‘I'm assuming Curley doesn't actually lick people and it's an expression, but there was an awful lot of them that went over my head.'*

*(P14N) ‘Early in this chapter the expression ‘rushing stars' made me question whether this was dialect and why the author has chosen this phrase.'*

Difficulties in becoming immersed centered upon feeling that the novel was unrealistic or through an inability to develop mental imagery.

#### Emotional Distancing

Where these difficulties arose, participants made surface-level appraisals about the novel within their diary entries. These appraisals included summaries of narrative events or attending to the stereotypes represented by the novel's characters and events:

*(P25A) ‘Lennie- seems like a stereotype of someone with a learning difficulty, like something out of an old film or tv show.'*

*(P6N) ‘some were one-dimensional i.e., the woman, Curley came across like a pantomime villain.'*

Surface-level thinking about the novel meant that participants remained within normative thinking processes, rather than exploring deeper meanings behind human emotions and social constructs. During these times, participants seemed to grow impatient with characters, showing frustration or annoyance toward difficult character behaviors that had culminated in emotionally difficult events within the novel. Rather than seeking to further explore these events and behaviors, participants tended to close down further opportunities to get inside the character's perspective as a defense mechanism:

*(P1A) ‘Annoyance at Curley's wife for not leaving Lennie be. She confided in Lennie that she had big aspirations and hated her husband, so she should have just divorced him and all of this could have been easily avoided.'*

*(P6N)'It made me angry because Curley's wife was racist, abusive and rude but got away with it because she was in a position of power.'*

For some participants their impatience toward characters continued into their writing, especially where participants were asked to write to a character:

*(P21A) [letter 2 self to Candy] ‘Candy— You're never going to see those rabbits, just because Lennie is dead. George will find a way to do it without you, but use all your money and possibly shoot you in the head.'*

*(P6N) [letter 2 self to Curley's wife] ‘making fun of a person because [of] race and disability is disgusting, it makes you a bully and a vile person, change the way you are and how you treat people or there could be consequences.'*

Here, ‘you're never going to see those rabbits' from P21A and ‘there could be consequences' from P6N pose threats to the futures of the characters that they are writing to. In this way, the participants' impatience for these characters had resulted in them simply ‘writing these characters off' in a way that closed down further empathic consideration.

#### Socio-Political and Historical Representation

When participants deliberately attempted to overcome their sense of disengagement, their efforts were often expressed through a socio-political and historical lens in place of in-depth feelings of personal involvement *with* the characters. This type of relatedness often resulted from general concerns across both groups with the racism, sexism, and classism within the novel. However, the autistic participants were additionally concerned with disability representation within the novel and surrounding concerns about ableism:

*(P27A) ‘And also, honestly, I wondered if the author just hates people with mental disabilities, or saw such a person like Lennie as some sort of literary device worth fetishizing rather than something that needed to be handled carefully in the literature.'*

*(P8N) ‘the continual negative descriptions of Curley's wife are noticeable. The only women described so far are her and talk about a brothel.'*

When operating from outside the text, participants often summarised these issues as easily recognized problems of the distant past, rather than as issues that are complexly bound into past and present human culture. This distance served as a way for some participants across both groups to emotionally remove themselves from the challenges of the content:

*(P27A) ‘In a modern context, maybe Lennie could have received the proper help and treatment, but in the 1930s, not so much.'*

*(P6N) ‘The old man was racist but it was a sign of the times and the south unfortunately'*

### Mobility of Response

#### Active Responding

One of the signs of immersion as compared to distance lay in participants' ability to move across the distances of the text itself, recreating the work's internal connections:

*(P27A) ‘[Lennie's death] It calls back to Candy's dog and Candy wishing he would've shot the dog himself because the dog was his responsibility; it's a harsher death for the dog to die at the hands of a stranger.'*

*(P8N) ‘A lot of this final chapter mirrors the rest of the book (repeating the dream, the shooting of Candy's dog, Lennie killing a small animal and grabbing a woman to feel the softness of their outfit). All of this was clearly deliberate.'*

As well as thinking across time and space, some participants additionally thought across multiple perspectives to gain deeper understandings. This was more common for the autistic participants:

*(P27A) ‘George felt responsible for Lennie and as much as I hate the author equating a handicapped man to a dog, I can see that same thought process going through George's head.'*

Here, P27A has overcome socio-political concerns by moving from the inferred perspective of the author into how the thought feels within the embodied perspective of the character George. Incidences of perspective mobility were especially prominent during the character letter task and, in one instance, the author letter task. Furthermore, perspective mobility was more prominent for autistic participants, who embodied character minds in a way that resulted in felt realism. Although non-autistic participants took character perspectives within their writing, the result was often more simplistic or hard to differentiate from the participants' own perspectives and tones:

*(P1A) [writing as Slim to George about him, Curley and Lennie] ‘I know that ain't none of your concern or fault as Curley showed you and Lennie no kindness and I don't blame you for getting the hell out of dodge but I was wondering if you'd have me over at your place. I worry that you or Lennie feel you could have stopped it but knowing Curley and how hot headed he was and the way his wife behaved…it was only a matter of time before something bad happened. But I'll do my part at your place, I think I can make a bit of business for us both by selling puppies to strangers and I know Lennie would be happy with a few around.'*

*(P14N) [letter 2 George to author] ‘At times I was mean to him, too, which I feel bad about because he didn't understand.'*

In P1A's character letter, he writes from Slim's perspective, aligning his writing with something of Slim's very tone and language, while also considering the perspectives of *both* George and Lennie. While P1A was the only autistic participant who chose to write from the perspective of another character, other autistic participants still addressed multiple character perspectives in their letters.

#### Thinking Aloud and Thinking Along

Participants who got inside the novel thought beyond the information that was overtly available to them. As a result, they remained open to alternative explanations of the same character:

*(P21A) ‘So I think the author was trying to make us see that Lennie is hopeless and George is So Good to Him but honestly I think there's something else going on that we haven't been told.'*

*(P10N) ‘I had mixed feelings about Carlson – was he being kind in putting an old dog out of its suffering? Or selfish as he didn't like the dog being in the bunkhouse?'*

As a result of this openness to alternative possibilities, sometimes expressed through questions, participants were then able to rethink their position as new information became available. This rethinking meant that participants engaged in live thinking within the ongoing processes of the novel, with the events of the story acting as a present reality to be continuously reassessed in real time:

*(P27A) ‘Seeing George somewhat portrayed as an unreliable narrator - so to speak - makes me wonder what else he could be lying about, specifically to Lennie, and if I need to rethink what his true intentions for and promises to Lennie could actually be. Something to keep an eye on.'*

*(P6N) ‘At first I thought the author was racist but the way he wrote about Crooks I have totally back tracked.'*

The use of ‘something to keep an eye on' here by P27A highlights the provisionality of thinking while reading, informed by the prior feelings of George being an ‘unreliable narrator.' By contrast, the ‘back tracking' from P6N goes beyond a change of mind, instead going back through the narrative to re-assess thoughts and feelings. While instances such as these occurred across both groups, autistic participants seemed more often to remain open to reassessments by thinking beyond the immediately available information.

#### Involuntary Feeling for

The more that participants had been able to successfully get into the novel, the more there were reports within participants' diaries of involuntary feelings for the novel and its characters. These involuntary feelings of creative discovery contrast to the earlier mentioned socio-political assessments that failed to get participants emotionally into the novel. In particular, the final two chapters of the novel often resulted in reports of overwhelming, involuntary sadness amongst participants:

*(P23A) ‘Sadness, resignation, fear of what would* ***happen*** *to the characters. I have a* ***sudden*** *feeling of terrible sadness about their dream of the farm, which I know* – ***and I think they know*** – *is too good to ever be true.'*

*(P10N) ‘Sadness – when dreaming about their future life – as it was far removed from their current situation'*

Here, P23A's ‘too good to ever be true' shows an emergent and involuntary saddened knowledge, rather than a cynical closing down of difficult feeling. Similarly, P10N's contrast between the dreams of the future to the present situation results in a wider and deeper understanding of the character's circumstances than they themselves have realised. Rather than this difference in understanding creating a distance between the reader and the characters, a painful knowledge results for the reader.

Where participants experienced these instances of painful knowledge, their emotions were not made any easier despite participant reports of knowing what was to come:

*(P11A) ‘Chapter 5 was a little bit like a car crash in slow motion, from the first couple of lines it's obvious what is going to happen'*

*(P17N) ‘The characters were eerily realistic'*

The obviousness described here by P11A is not a distanced knowingness but rather something that is felt painfully and sympathetically across the distance between P11A as the reader and the characters within the novel. P11A's metaphoric description of ‘a car crash in slow motion' shows this depth of empathy, felt across the gap between P11A's knowingness of what is to come and what the characters have yet to realise. These involuntary feelings were experienced by both autistic and non-autistic participants, but generally there was a sense that they appeared to be felt with greater depth by autistic participants.

#### More Than One Thing at a Time

Where participants had begun to successfully feel within the novel, there was a tendency to feel a greater complexity and register more than one thing at a time:

*(P27A) ‘Beyond that, this was a chapter I really felt like the characters were shades of gray.'*

This meant that participants also held in mind conflicting feelings toward characters, and non-conclusive views that further enhanced their willingness to actively rethink *while* reading in real time:

*(P1A) ‘George was harsh, more than once but I can* ***also*** *understand his frustrations with Lennie as* ***he*** *is* ***solely*** *looking after him and* ***they*** *seem to have run into trouble on more than one occasion due to Lennie's actions.'*

*(P8N) ‘George takes the role of a carer, who is exasperated and resentful at the difficulties in looking after Lennie*, ***but*** *obviously cares for him. I felt irritation at points, when he was being resentful toward Lennie*, ***but also*** *sympathy toward him, as it clear that Lennie's behaviour created patterns of difficulty across their lives.'*

Through P1A's move from ‘he' to ‘they' he expands upon his first thought of George being harsh by incorporating the realisation that George alone is responsible for what both he and Lennie go through together.

Through this willingness to hold in mind competing and even ambivalent views toward characters, participants were also able to feel for more than one character at a time. These instances remarkably included times where behavior of one character was itself not empathic toward the other characters in the novel, such that the reader even paradoxically tolerated intolerance:

*(P27A) ‘Even though neither Candy or Crooks showed her sympathy and even though she was expressing antagonism rather than vulnerability to match Crooks and Candy's antagonism, I was willing to sympathize with Curley's Wife.'*

*(P14N) ‘4 individuals can be so isolated, lonely and dependent even though they've been thrown together; that the differences between them (color, age, gender, ‘intelligence') can divide them despite them having so much in common; that they've all developed damaging self-protection mechanisms'*

For P23A, this feeling for more than one thing or person at a time led to her sense of feeling together with other readers:

*(P23A) ‘I was really sad that Lennie hurt the puppy. I knew he would. We all knew he would. He didn't mean to do it, but he did.'*

Here, the call from ‘I knew' to ‘we all knew' acts as a form of human understanding – a sense of true we-ness in human solidarity - holding together the difficulty of knowing that Lennie would hurt the puppy and feeling the painfulness of this for Lennie's sake too.

By thinking and feeling for more than one thing at a time, participants were then able to see deeper subtexts between characters. These assessments of subtext seemed more common and more in-depth amongst autistic readers:

*(P23A) ‘Lennie was killed at the time when he was gleefully recalling their dreams, their plans – the house, the rabbits, the alfalfa. With the shot to Lennie's head, George is also ‘killing' those dreams. He's killing that possible future, and I can't imagine he would want that same dream* ***without*** *Lennie there. The dream was* ***for the two of them, not for just one*** –*or for him and another.'*

*(P17N) ‘Lennie*, ***innocent but with*** *a power he couldn't control. Curley's wife craving attention* ***but unaware*** *of the dangers that flirting with Lennie could do'*

Through the breadth of understanding P23A is able to understand that George was also killing his own dreams in the death of Lennie.

#### Involvement in a Character

While these thoughts and feelings for more than one thing at a time led to a breadth of understanding, participants who got within the novel were also able to feel for the depth of a character by feeling with one character at a time in the midst of an interaction with another. Together with the breadth of feeling, this enabled the participants to further explore the subtext of the novel, particularly where characters had behaved in an unfavorable way:

(*P27A) ‘Even if he never truly expressed his love for Lennie beyond berating him every step of the way, there was a love there and there was a love in his final act of shooting Lennie.'*

*(P7N) ‘I felt sorry for Crooks as it is apparent he is isolated from everyone*, ***not just*** *the men on the ranch* ***but almost all*** *of Soledad'*

The metaphorical use of ‘killing' by P23A above in the ‘More Than One Thing at a Time' sub-theme. shows P23A taking on the novel's vocabulary to re-create the novel imaginatively. Comparatively, through deep understanding with George, P27A is able to see the love in the act of killing, an act that participants regularly believed had saved Lennie from an unkinder death at the hands of another character. It feels more paradoxical and more hard-won than a surface description of ‘mercy-killing.' This contrasts to the effortful implementation of socio-political empathy, where participants often centered their concerns around Lennie's death being painted as a mercy.

For autistic participants, but not non-autistic participants, this depth of understanding also applied to the character Lennie. Lennie has an unnamed disability, and his perspective comes primarily through the point of view of his non-disabled friend George and that of the other characters. However, autistic participants were able to use subtle cues in the text to infer for themselves Lennie's thoughts and feelings. While one non-autistic participant also briefly discussed Lennie's feelings, this was in contrast to what Lennie was *not* able to think and feel:

*(P14N) ‘Lennie's childlike happiness in hearing his favourite story…especially as a distraction from the fact that George should have been mad at him'*

*(P20A) ‘Lennie only feels shame, which shows that he does care about what he is doing'*

*(P23A) ‘I had great empathy for the ways in which Lennie was mentally beating himself up – saying cruel things to himself through imaginary people.'*

While autistic participants were familiar with considering different Others, it was the depth of feeling for the novel and its characters that prompted non-autistic participants to begin feeling for different Others outside of the text. In this way, the participants were more like a revised version of George. Specifically, it forced them to think about why Lennie was treated as an outcast by the other characters and ultimately unable to be accommodated:

*(P14N) ‘It challenges the reader to consider whether George had actually done the right thing and ultimately to ask why society was unable to accommodate Lennie.'*

*(P10N) [letter 2 self-author] ‘You have skilfully held up a mirror to society, which* ***many*** ***including myself*** *found uncomfortable when looking at its reflection. It made me reassess the world in which we live and what we as a society should be striving for. I* ***also loved*** *how there were so many characters who through no fault of their own were born or found themselves an outsider in an intolerant world (race, disability, poor) and yet many of these* ***outcasts*** *were* ***the warmest, kindest most decent*** *human beings within the book'*

These feelings, together with the in-depth feelings for Lennie from the autistic participants, contrast with the more generalised socio-political empathy relating to representations of disability. Those well-meaning general attitudes lacked this source-emotion to keep them fresh and authentic. Here, participants were able to feel for the ways in which human culture continues to make people unhappily Othered, whilst starting to unpick what creates this Othering.

### Re-creating Literature

#### Emotional Depth

Where participants were asked to re-write the ending of the book, the autistic participants in particular were able to draw on their thoughts and feelings as experienced from within the novel to re-create the literature in their own writing. Some of this ‘readerly imagination', infused with the language and feel of the book, has already been seen above in relation to sub-texts in the ‘Mobility of Response' theme. For non-autistic participants, this creation of a literary depth was only evident in creating emotional depth for George:

*(P1A) ‘A smile turns into unease, George tells himself “That son of a bitch and that harlot wife had it coming to them, to hell with them. I made it work Lennie, and I wasn't letting nobody stop me from living out our dream.” The sun sets, everybody heads in, life continues as normal.'*

*(P17N) [From Lennie's death]: ‘George felt something run across his leg. He looked down to see a pair of small, dark piercing eyes staring up at him. He stared back at the shapeless little face and stroked its back. “Come with me,” he whispered.'*

While P1A chose to undo the killing of Lennie, the result is not a mere escape from pain: the subtleties in his writing, starting with ‘a smile turns to unease', shows an understanding of how any ending would have led to emotional difficulty for the novel's characters. While P17N chose to leave the ending with the death of Lennie, the addition of George taking a rabbit with him shows a use of the novel's own materials in the partial compensation for the loss of companionship George felt in the death of Lennie, the rabbit standing in memory of Lennie.

Again, but now in their writing, autistic participants were equally drawn to Lennie's perspective in addition to that of George:

*(P23A) ‘He'd do it. He'd run away into the cave, and live there, no ketchup, just like he'd said.'*

*(P20A) “‘Listen Lennie, we ain't safe” “What you mean we ain't safe? We never safe George”'*

P20A's narrative is still shared between Lennie and George, as were her earlier considerations of character perspectives, adding a now shared knowledge for the precarious nature of their safety. P23A is able to re-use the novel's own language (‘no ketchup') to sustain Lennie's new but vulnerable independence.

Autistic participants were also able to use the differing perspectives of George and Lennie to build tension for their assumed readers. This again demonstrated mobility of perspectives for autistic participants—the perspective of two characters as perceived through the perspective of their audience:

*(P1A) ‘George walks up him, staring him in the eyes without blinking “Lennie, what did you do? You tell me now”*

*(P20A) “‘Yeah Lennie, you right, you right -ere”, George says as his voice begins to quieten down, into a soft whisper. “Why you whisperin'? I can't -ere you” Lennie says in normal volume.'*

The urgency created by P1A through George toward Lennie creates an elongated moment of tension where George does not yet know what Lennie has done wrong. In this way, the reader, who knows the events of the narrative, is left in suspense through various imaginative alternatives. Similarly, P20A, who previously demonstrated a depth of understanding for Lennie's perspective, here uses Lennie's lack of knowledge for the subtleties of the situation to build tension. In this exchange, readers are able to understand that George's whispering indicates the imminent threat to their safety, building tension around Lennie's lack of ability to understand this particular situation and respond appropriately with the same quiet urgency as George. P1A works through pace and timing; P20A through tone and volume. By such intuitively adapted techniques, autistic participants additionally incorporated the subtleties of human life that are often missed in everyday encounters, building upon the felt realism of the literature:

*(P23A) ‘He barely noticed breaking skin on his legs as he slipped on his way up over the rocks'*

*(P11A) ‘Despite being tired, the glimpse of their new home gave the men a renewed sense of energy, and had anyone been watching they might have said they moved a little faster and stood a little straighter.'*

#### Responsive Language Changes

Autistic participants further responded empathically by demonstrating responsive language changes, re-embodying the original novel tone through their own language choices:

*(P1A) ‘He heads over to Lennie, “What's got you all worked up now? You best not hurt that puppy none!”…“I done a bad thing George, but not that. I told her to stop screaming, but she wouldn't listen”.'*

*(P20A) ‘George held onto him tight and pulled Lennie in tighter, “Listen -ere, you gotta come with me right now Lennie, I ain't playin no games, none. We gonna be killed if we don't get outta here”*

*Lennie points to George's hand, “but you got that George, we safe”*

“*We ain't safe, I ain't even s'posed to have this thing -ere, it ain't mine, so we gotta go”*.

“*Well who's is it?” Lennie asks George, as if George was going to reply*.

“*Who's is it?”*

“*It ain't mine!”'*

In the movement of readers into writers through reading, a remarkable sustained empathy is created.

## Discussion

### Summary of Findings

The current study aimed to explore (1) the value of serious literature as a methodology for the exploration and comparison of autistic and non-autistic adult empathy and (2) whether adult autistic readers read in a more advantageous and empathic, ‘literary' way than non-autistic adult readers. Resultant findings are discussed below in relation to previous theoretical assumptions and associated findings.

#### Reading as an Advantageous Methodology for Empathy Research

Findings from the current study demonstrated the previously documented ability of serious literature to mirror the real social world (Mar and Oatley, 2008; Waytz et al., 2015; Oatley, 2016). While everyday socio-emotional encounters often require fast-paced assertions to achieve empathy, findings of improved empathic capacity after reading fiction (Mar et al., 2009; Bal and Veltkamp, 2013; Kidd and Castano, 2013) highlight the ability of literature to simulate everyday social cognition. Furthermore, participants in the present study demonstrated a felt realism for the literature with resulting experiences of embodied perspective and empathic engagement. These findings therefore support prior theoretical suggestions that literature promotes realistic feeling between the mind of the reader and the minds within the text in a way that results in character embodiment (Zunshine, 2011; Barnes, 2018; Mumper and Gerrig, 2019; Limburg, 2021). Additionally, these experiences of empathic embodiment created complex layers of thought together with feeling in a way that replicated the combination of affective and cognitive empathy as it is experienced within the everyday social world (Fletcher-Watson and Bird, 2020). In this way, the present study further demonstrates the advantages of serious literature as an ecologically valid tool within empathy research (Djikic et al., 2013; O'Sullivan et al., 2015; Chapple et al., 2021b). These advantages contrast to standardised ToM tests which instead seek to separate thought from feeling in an attempt to gain experimental control (Fletcher-Watson and Bird, 2020). Not only do such tests lack ecological validity, but they additionally favor simplistic, heuristic-based empathic assertions that prevent deeper empathic explorations (O'Sullivan et al., 2015; Fletcher-Watson and Bird, 2020). Given suggestions and findings that autistic individuals may be more socially tentative in their assertions (Capps et al., 1992; Murray et al., 2005; Milton, 2012, 2020; Chown, 2014; Lesser and Murray, 2020), standardised ToM tests therefore risk underscoring and subsequently underestimating the empathic abilities of autistic individuals. By contrast, the present study was able to demonstrate the complexity of the empathic responses experienced by autistic participants, who at no time demonstrated any specific empathy deficits when compared to non-autistic participants. As a result, the use of literature within empathy research poses an advantage in its ability to explore autistic experiences in a way that rehumanises understandings of autistic empathy by moving the focus away from what autistic people lack (Murray, 2020).

#### Addressing Theoretical Assumptions of Autistic Deficits

Overall, the multi-faceted empathic responses by autistic participants in the current study contest assumptions of an autism-specific empathy deficit (Baron-Cohen, 1997, 2002, 2009). Instead, autistic participants demonstrated reflexive thought alongside depth of feeling in a way that was empathic rather than systematic in nature, contrasting to the assumptions of the E-S theory (Baron-Cohen, 2002, 2009). Additionally, where perspective-taking and empathic feeling conflicted with autistic participants' own thoughts and feelings, they were able to draw from the novel's sub-text to overcome their own concerns. Therefore, findings challenge arguments that autistic individuals egocentrically impose their own thoughts onto the perspectives of others without regard to social context (Baron-Cohen, 1997; Lombardo and Baron-Cohen, 2011). These previous assumptions are instead likely to reflect the double empathy problem within research (Milton, 2012, 2020) alongside the overuse of restrictive cognitive ToM measures that prevent in-depth explorations of empathic experience.

Furthermore, autistic participants were able to think reflexively across the novel in a way that challenges the WCC theory's assumption of a resultant global processing deficit amongst autistic individuals (Happé, 1999). Similarly, autistic participants were more likely than non-autistic participants to think across perspectives within the novel. In this way, autistic participants demonstrated an ability to model minds, contesting the monotropism view that depth of feeling comes at the expense of perspective breadth (Lesser and Murray, 2020; Murray, 2020). However, the assumptions of the WCC and monotropism theories that autistic individuals have narrow interest systems which promote a depth of feeling and focus on detail were supported by the current research findings. Specifically, autistic participants demonstrated in-depth, involuntary feelings as well as a focus on subtle socio-emotional cues within the text which enabled them to uncover hard to reach perspectives. Therefore, findings suggest that an autistic neurocognitive advantage around depth of feeling may not result in deficits around breadth of understandings.

#### Double Empathy Implications

The ability amongst autistic participants to draw upon empathic depth alongside breadth often led to them demonstrating deeper feelings and understandings toward the literature than non-autistic participants. Specifically, autistic participants demonstrated more provisional thinking that enhanced their ability to hold in mind more than one conflicting mind or situation at a time. As a result, autistic participants were often more literary thinkers, able to ‘bite off more than they could chew', as required by the literature (Djikic et al., 2013; O'Sullivan et al., 2015; Davis, 2020; Davis and Magee, 2020). For example, where non-autistic participants were only able to use their creative writing to create emotional depth for the main character, George, autistic participants were able to model multiple minds, including harder to reach perspectives such as that of Lennie. Furthermore, autistic participants demonstrated abilities in embodying the language of the novel and drawing upon their literary reflections to re-create the literature in a way that captured the socio-emotional subtleties of character perspective and human feeling. The inclusion of these narrative features by autistic participants expands upon arguments that readers of serious literature ‘do' the literature in their reading (Barthes, 1969, as cited by Limburg, 2021; Muldoon, 2021) to suggest that autistic readers may engage more with literary thinking in this way. Overall, these findings support the double empathy problem assumption that autistic individuals may have more advantageous socio-empathic understandings than non-autistic individuals (Milton, 2012; Chown, 2014). Specifically, findings support the notion that, through their experience of navigating a lack of mutuality (Milton, 2012, 2020; Chown, 2014; Limburg, 2021), autistic individuals are more careful and provisional in their thinking and emotional responses (Capps et al., 1992; Lesser and Murray, 2020).

While the serious literature in the current study positioned autistic empathy as a social advantage, it further encouraged such tentative and provisional assertions amongst all participants. Early in the reading process, participants across groups tended to implement fast-paced, conclusive attributions of perspective that resulted in a failure to empathically get inside the literature. However, through literature requiring its readers to take on more than one thought and/or feeling at a time (O'Sullivan et al., 2015; Davis, 2020; Davis and Magee, 2020) participants were then required to go beyond heuristic-based assertions. While autistic participants were largely advantaged in this way of thinking, non-autistic participants began to think and feel for different Others throughout the novel. Furthermore, non-autistic participants began to re-think human culture by unpicking what creates Othering. These findings support previous findings that serious literature moves its readers away from rigid, stereotyped ways of thinking (Djikic et al., 2013; O'Sullivan et al., 2015). Additionally, the process of unpicking societal constructs indicates a potential for literature to give non-autistic individuals insight into the workings of wider society. In this way, literature may therefore be able to move non-autistic participants away from assumptions of pre-set mutuality and social norms (Milton, 2012; Chown, 2014) toward understanding the processes behind the Othering of neurodivergent individuals. Therefore, present findings indicate a potential for literature to promote double empathy understandings between autistic and non-autistic individuals, as shown in Chapple et al. (2021b), through a move away from assumptions of mutuality and pre-set social norms.

### Limitations and Future Research

The current sample consisted of participants who had all completed GCSE level education or above, with the majority of participants having completed degree-level education. This may have resulted from an increased willingness amongst individuals with higher education backgrounds to engage with serious literature. Furthermore, autistic participants were only included if they did not have additional disabilities that would affect their reading or writing skills. As a result, conclusions on the value of serious literature as a tool for exploring and comparing empathic experience is limited to the current sample and are not representative of the wider autistic community. Given the under-representation of autistic individuals with higher support needs within autism research, future research should seek to explore the value of reading and reflective writing in exploring the empathic experiences of autistic individuals from less educated backgrounds and with higher support needs.

Conclusions around autistic neurocognitive advantages in the contemplation of serious literature are also limited to the current sample. Although there was a spread of reader investment across neurotype groups, no data was collected on the wider reading habits of participants in the current sample. As a result, it could be that the autistic participants in the present sample were more experienced readers of serious literature compared to autistic individuals in the wider population of interest. Furthermore, that these participants were willing to engage in the reading of serious literature and subsequent reflections may have reflected an increased ability and willingness for reflexive and tentative thinking amongst these participants. Additionally, while *Of Mice and Men* (Steinbeck, 1937) was chosen due to its representation of adversity and ableism, this increased relevance for autistic participants may have shaped their responses in a different way than the non-autistic participants within the sample. As a result, conclusions around deeper empathic understandings amongst autistic individuals are limited to both the current sample and the piece of serious literature. Therefore, future research should seek to compare the reading experiences and reflections of autistic compared to non-autistic adults in response to various text types with different content relevance. Additionally, further enquiry is needed to explore specific textual factors, such as genre and realism, that enhance double empathy understandings and the ability of autistic readers to get emotionally inside a text.

### Conclusion

In conclusion, the findings of the present study demonstrate the utility of serious literature as a research tool for exploring empathic experiences between autistic and non-autistic individuals. Furthermore, the implementation of serious literature in the current study was able to demonstrate the complex empathic experiences of the autistic readers within the study. Importantly, these findings contest previous assumptions of an empathy deficit amongst autistic individuals (Baron-Cohen, 1997, 2002, 2009) as well as assumptions of an autistic deficit in the modeling of other minds (Baron-Cohen, 1997; Happé, 1999; Murray, 2020). Instead, findings supported previous suggestions that autistic individuals are more socially tentative (Capps et al., 1992), feeling with others with advanced depth (Murray et al., 2005; Lesser and Murray, 2020; Murray, 2020) and with provisional assertions. As a result, the present findings support the notion that, possibly through their experience in navigating a lack of mutuality, autistic individuals have enhanced socio-emotional understandings that can prevent socio-communicative breakdowns (Milton, 2012, 2020; Chown, 2014). However, findings from the current study indicate that serious literature may encourage similar provisional assessments and socio-empathic understandings amongst non-autistic readers. Therefore, these findings demonstrate the full potential of serious literature to promote double empathy understandings amongst autistic and non-autistic individuals, to break down barriers and to advance a more nuanced scientific study of autistic psychology.

## Data Availability Statement

The datasets presented in this article are not readily available because the raw data is ethically sensitive, in that participants could possibly be identified from their data. Requests to access the datasets should be directed to Melissa Chapple, melissa.chapple@liverpool.ac.uk.

## Ethics Statement

The studies involving human participants were reviewed and approved by University of Liverpool Ethics Committee. The patients/participants provided their written informed consent to participate in this study. Written informed consent was obtained from the individuals for the publication of any potentially identifiable images or data included in this article.

## Author Contributions

MC, PD, JB, and RC: conceptualization, methodology, and validation. MC: funding acquisition, data curation, project administration, and writing—original draft. MC, PD, JB, SW, and RC: formal analysis and writing—review and editing. PD, JB, and RC: supervision. All authors contributed to the article and approved the submitted version.

## Funding

This research was funded by an Economic and Social Research Council Doctoral Studentship.

## Conflict of Interest

The authors declare that the research was conducted in the absence of any commercial or financial relationships that could be construed as a potential conflict of interest.

## Publisher's Note

All claims expressed in this article are solely those of the authors and do not necessarily represent those of their affiliated organizations, or those of the publisher, the editors and the reviewers. Any product that may be evaluated in this article, or claim that may be made by its manufacturer, is not guaranteed or endorsed by the publisher.
